# Food and nutritional security of semi-arid farm families benefiting from rainwater collection equipment in Brazil

**DOI:** 10.1371/journal.pone.0234974

**Published:** 2020-07-14

**Authors:** Andhressa Araújo Fagundes, Tatiana Canuto Silva, Silvia Maria Voci, Fernanda dos Santos, Kiriaque Barra Ferreira Barbosa, Ana Maria Segall Corrêa

**Affiliations:** 1 Graduate Program in Nutrition Sciences, Federal University of Sergipe, São Cristóvão, Sergipe, Brazil; 2 Department of Nutrition, Federal University of Sergipe, São Cristóvão, Sergipe, Brazil; 3 Graduate Program in Collective Health, Faculty of Medicine, University of Campinas (Unicamp), Campinas, São Paulo, Brazil; National Institute of Health and Nutrition, National Institutes of Biomedical Innovation, Health and Nutrition, JAPAN

## Abstract

The objective of this study was to identify and describe the experience of family farmers and their respective families after using the Boardwalk Cistern rainwater collection system and consequent impacts on nutrition profile and food security. This is a qualitative-quantitative study conducted in two municipalities in the semi-arid region of the state of Alagoas, northeastern Brazil. A structured questionnaire was applied to collect information on demographic and socioeconomic status and household access to food, based on the Brazilian Food Insecurity Scale of 29 family farmers’ households. Food intake was assessed by food intake markers of the Ministry of Health, while nutritional status was determined by measuring the weight and height of all family members and waist circumference of adults. Nutrition diagnosis was performed using the cutoff points of body mass index for age. Three focus groups were conducted, and the information collected was analyzed through Content Analysis with the aim of knowing the participants' perception of the effects of the received water equipment. The study showed a high prevalence of excess weight (52.7%) and high risk for cardiovascular diseases (35.9%) marked by a high salt and sugar in the food intake. Food Insecurity Scale showed that food insecurity is a problem occurring in 75% of these families. However, focus groups showed that families have a positive perception of Boardwalk Cisterns for their food security. They believe that agricultural production has improved, thereby offering a wider range of foods and, consequently, improving food security. In conclusion, this study highlights the importance of water access programs for food production within public policies to guarantee FNS.

## Introduction

Previous research has shown that food insecurity is more common in rural areas compared to urban areas [1,2]. In Brazil, this situation can be explained by the lower education and income level of residents of rural areas, as well as the difficulty of access to water, land, and food production [3–6]. Access to water is one of the guidelines of Brazil’s Food and Nutritional Security (FNS) policy, especially for people who face water scarcity and for family farmers [7]. It is a government policy to be supported by funds provided for in the Federal Government’s budget. Through the "Cisterns Program", the Brazilian government has installed more than one million cisterns throughout the Northeastern region since 2003. This program is mainly targeted at low-income rural families living in places affected by drought or natural water scarcity, with priority for traditional people and communities [8], e.g., indigenous people, “quilombolas”, rubber tappers, riverine gatherers, “mangaba” pickers, babassu coconut breakers, and others. The goal of the program is to provide access to water for human consumption and food production through the implementation of social technologies.

This condition has raised research issues that guided this study. What is the food and nutrition security situation experienced by families that use the Boardwalk Cistern rainwater collection system, aimed at increasing food production by family farmers? Have the lives of families and their FNS situation improved after the implementation of the Boardwalk Cistern system? How do families perceive the effect of cisterns on their lives? Do they continue to experience situations of vulnerability and food and nutrition insecurity?

FNS is defined as the fulfillment of everyone's right to regular and permanent access to sufficient and nutritious food, without compromising access to other essential needs [7]. This concept goes beyond the quantitative and biological aspects of eating; it also involves food quality, environmental conditions for food production, sustainable development, quality of life, and access to water [9,10].

An approach to a multifaceted situation, influenced by several factors, as is the case of FNS, requires the use of different evaluation and monitoring methods [11]. Research on food intake is one of the ways to detect food and nutrition insecurity since it offers detailed and valuable data on food intake [12]. Another method of indirect FNS analysis is the anthropometric assessment of individuals, which measurements provide important indicators of nutritional status that, in most cases, are impacted by the FNS status of families [13].

There are few studies published in Brazil on the evaluation of FNS and related programs. Usually, they are either institutional publications or research conducted by ministerial offices of the federal government responsible for initiatives aimed at benefiting this population.

Therefore, the objectives of the present research are twofold. Firstly, to understand FNS as experienced by family farmers (and their families) who use the Boardwalk Cistern rainwater collection system in two municipalities of the semi-arid region of the state of Alagoas, Northeastern Brazil. Secondly, to identify how they perceive the effects of this system on their lives.

## Methods

This qualitative/quantitative study was carried out with data collection through a household survey and three focus groups. The mixed-methods approach was adopted for triangulation since it enables a clearer understanding of the phenomenon. It involves a correlation analysis of results from different sources based on the same object [14].

This study is part of a significant research project of the Brazilian Agricultural Research Corporation (Embrapa) regarding rainwater collection and management for food production [15]. Such a project was carried out to share knowledge and technologies for water collection, storage, and management in rural communities that are aided through development-oriented initiatives and policies of the "Brazil Sem Miséria" (Brazil Without Misery). This Program was created to overcome extreme poverty in Brazil, focusing on all the dimensions that poverty manifests itself, organized in three axes: income guarantee, access to public services, and productive inclusion [15]. An action plan was designed for each territory based on demands arising from meetings held in Brasília-DF and Petrolina-PE. The main project was started in the semi-arid region of Alagoas in 2013. Families were included in the present research if they had benefited from the social technology for rainwater collection called Boardwalk Cistern, offered by the “Brazil Sem Miséria” Program, in 2013.

**Fig 1:**
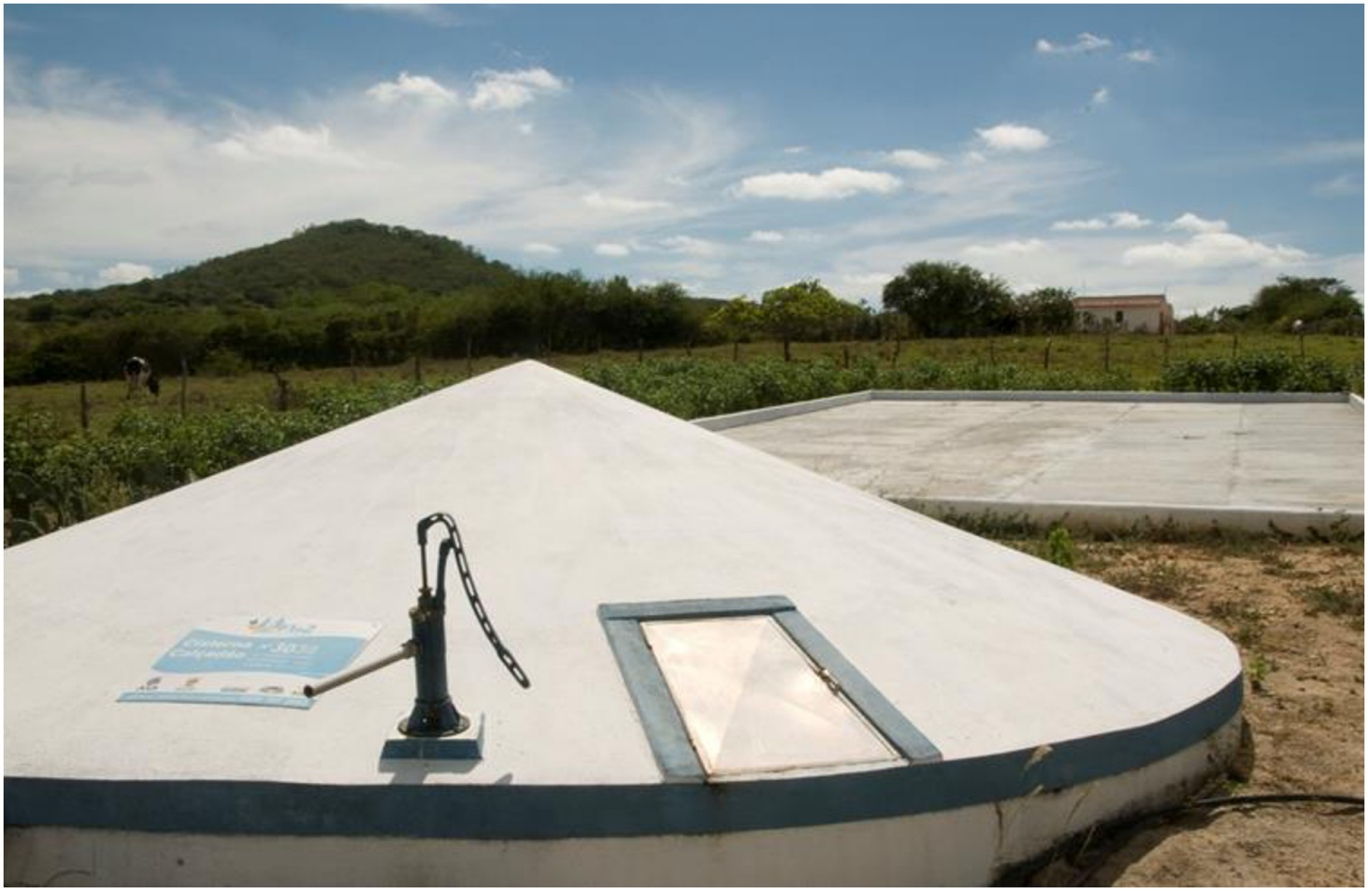
Boardwalk Cistern, a 52,000 liters water tank connected to a boardwalk that captures rainwater [16].

In this sense, the sample used in this study consisted of the same sample assessed in 2013 in the first research project. Data collection was performed between June and November 2016, as a moment of reassessment of these families. The sample composed of 29 households of family farmers selected in the municipalities of Igaci and Craíbas, in the semi-arid region of Alagoas, Brazil, out of a total of 30 households that had received the Boardwalk Cistern program (Fig 1) in that region.

Data collection instrument consisted of a questionnaire on demographic and socioeconomic status, living conditions after the implementation of cisterns, as well as other aspects such as food produced for consumption and marketing purposes, and main initiatives undertaken by households in the absence of food and food consumption. The data collection questionnaire was mainly developed for this purpose, based on the questionnaire of the National Household Sample Survey [17].

To identify situations of household food security, the Brazilian Food Insecurity Scale was applied; particularly, the version by National Household Sample Survey 2013, since it is widely used by the Brazilian Institute of Geography and Statistics in urban and rural environments [18]. This scale is the instrument most often used for an accurate evaluation of vulnerability regarding food and nutrition of families [10].

Food intake was assessed using food intake marker forms from the protocol of the Food and Nutrition Surveillance System, as recommended by the Ministry of Health [19], in order to complement information collected in the questionnaire, such as: having meals watching TV, working on the computer and/or cellular phone; meals taken throughout the day; food consumption from the previous day. This form was applied to all participants over two years of age present at the household, and answered by the family heads.

Nutritional status was assessed based on the weight and height measurements of all family members present at the moment of data collection, and waist circumference was assessed only in adults. Diagnosis used the cutoff points recommended by the Ministry of Health for Body Mass Index (BMI) for age [20].

Data were collected by researchers from Embrapa and the Association of Alternative Farmers (AAGRA), as well as by two teachers, a master's student and four undergraduate students from the Federal University of Sergipe, who were previously trained to collect data during household interviews.

Data were analyzed using the *Statistical Package for Social Sciences* (SPSS), version 17 for Windows. Numerical variables were presented as mean and standard deviation and categorical variables as simple and relative frequencies. Inferential statistics were performed using Pearson's chi-square test and, when applicable, Fisher's Exact Test to check for associations among categorical variables.

To understand participants' perception of the effects of Boardwalk Cistern on their lives, three focus group sessions were conducted with a representative of each family participant at the stage of data collection. Each session lasted for approximately 60 minutes. The focus group technique offers insights into participants' reactions, and perceptions of actions and processes and makes researchers more aware of the needs of study groups [21]. All participants were identified with a name tag and sat in a circle. This way, they could face one another, which encouraged interaction in the debate. This technique is characterized as a small, informal discussion where issues should be following participants' intrinsic practices and concepts, in this case, farmers, in order to obtain an in-depth qualitative information. The methodology suggests groups that share similar characteristics to be given the best interaction to investigate the theme [14,22].

In this study, sections were held at the community association poles, in neutral places, preserved from interruption and noise, and families were invited according to their proximity to each pole. Focus groups were conducted by a moderator whose role was to assist the discussion by asking the initial and other periodic questions, to balance the responses of the most extrovert members, and to encourage the participation of the introvert members, and an observer [21]. The sessions were conducted on a previously elaborated script and was carried out by Embrapa's researcher and the nutrition teacher.

The script in use contained the following questions: (a) 'What has improved in your lives in the last few years?'; (b) 'How does the cistern help you/facilitate your life?' (c) 'What was your life like before using the Boardwalk Cistern system?'; (d) 'Do you have any problems when using the cistern?'; Moreover, (e) 'As a group, how do you think you can make better use of the water collected in the cistern for food production and rearing of small animals?'. Data collected were analyzed through the Content Analysis technique, which is defined as a set of research techniques whose objective is to clarify meanings contained in the text (semantics) [23–25].

The Research Ethics Committee approved this study of the Federal University of Sergipe. It followed guidelines of Resolution 466/2012 of the National Health Council. All participants signed an Informed Consent Form authorizing the use of voice and image data.

## Results and discussion

A total of 113 individuals participated in the survey, who belonged to 29 families that used the Boardwalk Cistern system; 71 of them were present at the time of questionnaire application. Data on dietary intake and nutritional status correspond to all individuals present in the household at the time of collection and who agreed to participate in the research. Family farmers, who were the heads of their families, were the primary respondents of questionnaires and the Brazilian Food Insecurity Scale. Results were presented per the research phase, and the population assessed.

### Phase 1—Sample characterization

#### Farmers (family heads)

Out of the total number of family heads (n = 29) who participated in the study, 44.8% were females. This rate is slightly higher than the proportion found nationwide [26] and similar to the mean rate found in Northeastern Brazil (42.9%). The results of the National Household Sample Survey, held in Brazil in 2013, indicated a more marked increased prevalence of Food Security (FS) in households whose heads were females: 65.6% in FS and 14.2% in situation of moderate or severe Food Insecurity in 2009, compared to 74.6% in FS and 9.3% in moderate or severe Food Insecurity in 2013. Despite the significant increase of FS in this period, the prevalence in 2013 is still lower compared to households whose heads were males: 79.1% [18,27].

When asked about the main purpose of using the cistern, a large part of the respondents mentioned more than one possible use. Clearly, they used water from the cistern in a different way from that recommended by the "Uma Terra, Duas Águas” Program (One Land and Two Waters Program) [8,28], that provides one land and two water: one for planting, another to raise small animals; 82.8% mentioned that they used it for food production, 69.0% for consumption, 55.2% for hygiene and 44.0% for animal rearing.

Regarding cistern operation, 89.7% of respondents answered that 'it worked well'. Three (10.3%) reported that the cistern did not work correctly, corroborating a study conducted by Fonseca [29], which found that 13.3% of participants reported that their cisterns leaked immediately after construction. Data indicate that families had a positive overall perception of the cistern operation, and such perception was not negatively affected by cisterns that did not work properly, e.g., those with cracks and leaks.

Based on the application of the Brazilian Food Insecurity Scale, 62.9% of families had members under 18 years of age. There was high prevalence of food insecurity among participants: 79.1% presented food insecurity (FI); 28.1% of them were classified as having mild FI, i.e., concern or uncertainty about access to quality food in the future in adequate amount; 26% showed moderate FI, i.e., consumption of lower amounts of food and/or disruption of eating habits as a result of food scarcity among adults; and 25% showed severe FI, consumption of lower amounts of food and/or disruption of eating habits as a result of food scarcity among adults and/or children, and/or hunger [18,30].

The prevalence of food insecurity in this study was higher than that found by Rocha et al. [31] in families in rural communities living in the semi-arid region of the state of Ceará, namely, 33.2% with mild FI, 17.8% with moderate FI and 7% with severe FI, reflecting an unfavorable scenario even after the intervention of cisterns.

Despite the high FI rate in the study population, a large part of family farming is focused on agroecology, which is one of the ways to ensure FNS. Machado [32] argued that agroecology should not be merely applied as a production technique; but should be linked to social, political, economic, administrative, energy, environmental, cultural, and technical dimensions. Otherwise, it will turn out to be a conventional technique. This author believes that producing food is not enough; such production has to respect nature and protect biodiversity by including the dimensions mentioned above. As a result, nutritional quality can be enhanced through more sustainable production practices, which can also increase access and availability of food.

Regarding the strategies used by families to deal with food insecurity, it was found that 17.4% of farmers reported food scarcity over the last three months. When faced with food scarcity in the household, 10.3% of farmers answered that they ‘bought food on credit’, as a strategy. The same attitude was reported as the primary strategy adopted by families (43.3% of households) when facing food scarcity, according to a survey conducted nationwide [33].

Statistical analysis showed association for two variables: 'Food scarcity over the last three months' and 'Presence of fruit trees in the backyard' (mango, acerola, pine cone and guava). The analysis showed that, of all respondents who reported no food scarcity over the last three months, 88.5% had trees in their backyards (p = 0.026). Data have shown that something commonplace and straightforward, e.g., presence of fruit trees in the backyard, can lead families (such as those participating in this study) to have a more positive perception about their food security situation.

The Boardwalk Cistern system fulfills this requirement of ensuring FNS, enabling farmers to achieve greater food production [34]. In fact, data found in this study showed that all respondents reported changing their eating behavior after the implementation of the Boardwalk Cistern system, corroborating findings of de Fonseca [29]. Farmers reported that in rainy years, when rainfall is below average values, the size of the ground-level catchment platform ensures that the cistern is filled, thus allowing irrigation of vegetable gardens, seedlings, fruit trees, "mandala" gardens, or use of water for the raising of small animals (chickens and bees). Therefore, the FNS situation for families is improved. The changes cited by farmers surveyed include the development of vegetable gardens and greater variety in the supply and consumption of pesticide-free foods–factors related to the consumption dimension of FNS.

Out of the total number of farmers surveyed, 96.6% reported greater product diversification and improvement, and 72.4% reported an increase in their income, which exemplifies how the Cistern Program worked as a tool for promotion of Human Right to adequate food, FNS, and food sovereignty. The concept of food sovereignty legitimates that each nation has the right to define policies that guarantee the FNS of its population, including the right to preserve traditional production and food practices. Moreover, it is essential to state that such a process must take place on a sustainable basis from the environmental, social, and economic point of view [35].

#### Family

Table 1 shows information about the other household members who participated in the survey: a total of 113 individuals, including participants who were present at the time of the survey plus members of families whose data were reported by the respondent. The average number of individuals per household was 3.9 (±1.87). A similar result was reported by Rocha et al. [31], who found that families living in the semi-arid region of Ceará had 4.2 residents, on average. The National Household Sample Survey (2013) reported that the higher the number of household residents, the lower the prevalence of FNS [18].

**Table 1:** Socio-demographic characteristics of the population in the semi-arid region of Alagoas, Brazil, 2016 (n = 113).

Variable	n (%)
Age	
Children (< 10 years)	13 (11.5)
Adolescents (≥10 years and < 20 years)	24 (21.2)
Adults (≥20 years and < 60 years)	68 (60.2)
Elderly (< 60 years)	6 (5.3)
No answer	2 (1.8)
Gender	
Females	57 (50.5)
Males	56 (49.5)
Kinship relation with the family head	
Head	29 (25.7)
Spouse or Partner	27 (23.9)
Child	48 (42.5)
Other kinship relations ^(^^i^^)^	9 (8.0)
Enrolled in schools	42 (37.2)
Knows how to read and write	85 (75.2)
Work status	
Farm Worker/Farmer	61 (54.0)
Retired/Pensioner	4 (3.5)
Unemployed	11 (9.7)
Other ^(^^ii^^)^	22 (20.1)

^i^ Grandson/great-grandson, son-in-law/daughter-in-law.

^ii^ Other: Employer, the wage-earning worker with formal work contract; wage-earning worker without formal work contract; temporary work; and unemployed.

Regarding food intake, a relevant fact was the high sugar consumption monthly, according to the information collected by the questionnaire. Households consume an average of 9.1 (±6.4) kilograms of sugar per month. Considering an average of 3.9 (±1.87) persons per household, sugar intake is equivalent to 76 grams/person/day. In 2015, the World Health Organization (WHO) published guidelines on sugar intake for adults and children, stating that the consumption of simple carbohydrates should not exceed 25 grams per day [36].

The average monthly salt intake of households is 1.3 kg (±0.87), which results in the consumption of 11 grams/person/day. WHO recommends that salt intake should not exceed 5g/ day. Respondents also reported an average monthly oil consumption of 3.4L (±2.4), which is high and equivalent to 29 ml/person/day. The Dietary Guidelines for the Brazilian Population advise that oils and fats should be consumed in small quantities [37].

Among household (n = 64) food consumption markers (Table 2), food groups most consumed by families on the previous day were beans (93.8%), followed by vegetables and legumes (65.6%). In contrast to these positive results, they reported a high percentage of consumption of sweetened beverages (65.6%) and low consumption of fresh fruits (40.6%). Also, they often mentioned the consumption of ultra-processed foods, e.g., instant noodles, processed snack foods, sandwich cookies/sweets or treats, hamburgers, and sausages.

**Table 2:** Frequency of eating behavior and dietary intake of the study population. Semi-arid region of Alagoas, Brazil, 2016 (n = 64*).

Variable	n (%)
Having meals watching TV, working on the computer and/or cellular phone	14 (21.9)
Meals eaten throughout the day	
Breakfast	62 (96.9)
Mid-morning snack	28 (43.8)
Lunch	61 (95.3)
Mid-afternoon snack	28 (43.8)
Dinner	58 (90.6)
Supper	15 (23.4)
Food eaten the day before	
Beans	60 (93.8)
Fresh fruits	26 (40.6)
Vegetables/Legumes	42 (65.6)
Hamburger and/or sausages	13 (20.3)
Sugar-sweetened beverages	42 (65.6)
Instant noodles, processed snack foods or salty cookies	21 (32.8)
Sandwich cookies/sweets or treats	18 (28.1)

* Valid answers for consumption marker variables.

Although they reported eating vegetables and legumes, they consumed sweetened beverages at similar levels. Such consumption was considered to be quite high, given the excess of pure sugar, in particular. The population presents excess weight and high cardiovascular risk, which is aggravated by the consumption of simple sugars above-average levels, according to the data presented above.

Importantly, although family farmers grow fruits, such as mango, acerola fruit, and guava, processed sweetened beverages are consumed more often than natural juice. Similar results were reported by the 2002–2003 and 2008–2009 Household Budget Surveys; there was a decrease in the consumption of fruits and vegetables, which became insufficient, as opposed to the presence of excess calories from sugar and saturated fat in the diet of Brazilians [3].

The findings of these studies are similar to those by Carvalho and Rocha [38], which showed that although respondents have more access to this food group, consumption is low. This can be explained by several factors: for example, the production of these foods is probably intended for marketing rather than for household consumption. Influenced by technological advances in the food industry, agriculture, and the globalization of the economy, dietary behavior has been a matter of concern for health sciences since epidemiological studies have signaled a close relationship between the high-energy, high-fat diet. And in simple carbohydrates and some non-communicable chronic diseases [39]. Lack of time was also one of the factors that influenced the significant changes in eating behavior, increasingly imposing a fast and practical diet having to resort to ultra-processed ready to eat foods [40]. Another possible reason may be due to issues related to eating behavior.

Brazilian national data showed that the indicator of malnutrition, the height deficit, decreased from 29.3% (1974–75) to 7.2% (2008–2009) among boys and from 26.7% to 6.3% in girls. They also point out that overweight boys aged 10–19 years increased from 3.7% (1974–75) to 21.7% (2008–2009). Among girls, the growth of overweight went from 7.6% to 19.4%. This shows that malnutrition in Brazilian children decreased and the overweight and obesity increased [3,27].

Children and adolescents have had increasingly early access to high-fat, nutritionally deficient diets and eat small amounts of fruits and vegetables. Besides, hours spent on sedentary activities such as watching television, using mobile phones and computers, and others are more closely related to the development of obesity [41]. There is evidence that the situation experienced at the time of making meals (e.g., eating while watching TV, alone, or while sitting on the couch; or sharing a meal and sitting at the table with other people) is essential to determine which foods are to be consumed and, more importantly, the amount of food to be consumed [37,42]. These consumption characteristics may have a direct relationship with the nutritional status found in this population.

At the time of the survey, 71 individuals (62.8% of the total number of members of all families), were assessed for nutritional status. In the adult population, the body mass index (BMI) showed that 41.8% had average weight, 32.7% were overweight, 20% were obese, and 5.4% had a low weight. There was also a high percentage of risk for cardiovascular diseases (35.9%), according to waist circumference measurements.

Even though there was high percentage of excess weight (52.7%), results found for farmers were lower than the prevalence found by the Family Budget Survey (2008–2009) in an anthropometric assessment conducted in households in all regions of Brazil, which reported that 57% of people had excess weigh in rural communities of the northeastern region [3]. Among adolescent and elderly patients assessed, there was 50% excess weight and 50% average weight; 75% of children showed appropriate BMI for an age while 25% were overweight, according to the same index.

In a study assessing the nutritional status of children and adolescents of a municipality in the semi-arid region of northeastern Brazil conducted by Ramires et al. [41], prevalence of 24% of overweight children and adolescents was found, which reflects the characteristic of a complex epidemiological profile arising from nutritional transition, caused by the double burden of diseases on both sides of food insecurity- malnutrition and overweight.

National data have shown that the prevalence of excess weight increased in the Northeastern region of Brazil, which is worrying because it is related to the development of cardiovascular diseases. The Family Budget Survey 2008–2009 pointed out that malnutrition during the first years of life and excess weight and obesity in other age groups are major public health problems in Brazil [3,43,44].

According to some studies [27,45–47], excess weight has been increasingly significant in the most disadvantaged socioeconomic classes severely affecting children, especially those under two years of age, whose the authors claimed that poor nutrition is the main trigger of excess weight (95%), while endogenous causes account for only 5%.

The nutritional status of family farmers in this study is similar to those described in the study by Oliveira et al. [48]). They found that overweight was predominant, reflecting a change in Food Insecurity status, previously characterized as malnutrition after an assessment based on nutritional status.

In another study conducted by Hernandez et al. [49] aimed at assessing whether there are differences based on gender and race/ethnicity between food insecurity and the overweight/obesity relationship among adults aged 18 and 59 years old. The results indicated that there were differences according to gender and race/ethnicity in terms of food insecurity overweight/obesity. Several studies on food insecurity and obesity found a positive association, especially among women [49–51]. The main points presented in these studies showed a higher percentage of women with food insecurity were overweight compared with women with food security, among all ethnic groups, confirming that food insecurity and the obesity remain firmly and positively associated with women, and food insecurity is more common in young adult women (14%) than young adult men (9%).

The dietary profile of farmers in these studies may be linked to data on overweight and risk for the development of cardiovascular diseases found in the present study.

### Phase 2—Qualitative

The hierarchical classification of data collected through the Focus Groups resulted in the *corpus* that was analyzed, and the content of the first two issues was organized into four categories. Table 3 shows data on the importance of Cisterns for study participants.

**Table 3:** Categories of families' perception of what has changed in their lives in recent years and how the cistern helps them. Semi-arid region of Alagoas, Brazil, 2016.

Category	Percentage rate
Food production	41.6% (15)
Healthy eating and health	33.3% (12)
Positive feelings	33.3% (12)
Better living conditions	25.0% (9)

‘Better living conditions’ category is about changes that occurred in the lives of individuals, e.g., the dynamics of fetching water after traveling many miles.

Table 4 shows previously presented categories and some of the quotes of participants during the Focus Group about the importance of cisterns (questions 1 and 2 of the script).

**Table 4:** Families' perception of what has changed in their lives in recent years and how cisterns have facilitated their lives in the semi-arid region of Alagoas, Brazil, 2016.

Category	Quotes
Food production	*"I can have my little vegetable garden now*. *I could not have it before*, *in the summer"*. *"I am growing all sorts of things*: *boldo*, *lettuce*, *onions*, *cucumber*, *eggplant*, *beet*. *They are all growing well*, *thank God* …*"*
Healthy eating and health	*"Good health*! *Besides nutrition*, *there is health*, *right*? *You go to the orchard in the morning*, *and you fetch papaya like this*, *you cut it*, *and then I can say that I have planted it*, *you see"*. *"If it were not for the* Boardwalk Cistern, *we would not be eating fresh vegetables* …*"*
Positive feelings	*"It was a blessing* … *My cistern is there*, *and I can collect water*, *thank God"*. *"It is the best thing I have ever had in my life* ….*" "I cannot be thankful enough for this gift* … *I am very happy to have it or else things would be worse"*.
Better living conditions	*“Our life is a lot better*. *We had to go fetch water wherever we could find it* … *Now we no longer need to carry water around”*. *"We have water near our home*, *so we do not have to go fetch it*, *and we do not have to pay someone else to do that*, *either* … *right*?*"*. *“Water means life*, *you know*?*”*

The Ministry of Social Development conducted a qualitative assessment of the “Uma Terra Duas Águas” Program, with 41 families living in the states of Bahia, Sergipe, and Rio Grande do Norte [52]. The results showed several positive factors (e.g., improved access to water, which reduced the time spent to travel to other places where water was available). Also, increased planting of vegetables and fruit trees was observed, resulting in higher consumption and variety [52,53]. The present study differs from the study by the Ministry of Social Development in 2011 [52] since it included quantitative research in addition to the qualitative approach. Thus, it offers further insights into the FNS situation of participating families.

Table 5 shows categories of participants' perception of what life was like before the Boardwalk Cistern: 'Limited fresh food intake', 'Storage', and 'Obstacles'. The first category, 'Limited fresh food intake', refers to the food dimension present in the FNS concept, i.e., food production and availability should be enough and adequate to meet the needs of individuals both in terms of quality and quantity [34]. ‘Storage’ is the second category, which refers to problems faced by families for storage of the small amount of water that they could access. The last category, ‘Obstacles’, refers to challenges faced by families as a result of water scarcity.

**Table 5:** Categories and quotes regarding the perception of what life was before the Boardwalk Cistern. Semi-arid region of Alagoas, Brazil, 2016.

Category	Quotes
Limited fresh food intake	"We could only buy food by the kilo. It was all of the same type". *"I would only buy vegetables when I had the cash to spare"*. *" [*.…*] many types of orange*. *We only had one type*, *you know*?*"*
Storage	*"I bought the barrel*, *but where could I store water*? *There was nowhere I could put it in; we could not buy two*, *three barrels"*. *"I did not even have a bucket to put water in*. *Before*, *I had to use up the water because I could not store it"*.
Obstacles	*“I had to fill up some many barrels that I now got a herniated disc*. *Water barrels are heavy”*. *“It was only one water barrel* … *no plantation grew* …*”*. *“I traveled up to five times to fetch water for my animals”*.

## Conclusions

Families in the present study usually experience both food insecurity (due to the difficulty of accessing adequate food) and nutrition insecurity (as a result of the high prevalence of excess weight in the study group). Therefore, different types of actions are needed to promote FNS, e.g., improve people’s income and education to increase their autonomy and, thus, help them make better food choices.

Nevertheless, it could be concluded that these families have a positive perception of the Boardwalk Cistern system. They reported several improvements that occurred after the equipment was installed, for example, the intake of better and more diverse fresh food. Regarding food intake, there is a low consumption of fruits. Instead, they consume processed products, especially sugar-sweetened beverages. Although such consumption is low, reduced intake of fruits and vegetables is the primary strategy adopted by families to deal with food scarcity.

Finally, another conclusion is that social technology, as represented by the Boardwalk Cistern system, improved production and access to food, including fresh food. Programs for access to water for food production purposes, within public policies, are crucial to ensure FNS. Moreover, these programs should increase the food and nutrition education of this population. One limitation of the present study is the absence of a comparison group, e.g., families who have not received the Boardwalk Cistern system.

## Supporting information

S1 File (ZIP)
